# Spinal Injuries

**Published:** 1889-11-23

**Authors:** 


### SURGERY.

### SPINAL INJURIES.

Dr. F. X. Dercum, of Philadelphia, has some remarks in a late number of the *Therapeutic Gazatte* on "Spinal Injuries," more especially railway spine. The term railway spine is regarded as objectionable, as the expression implies there is something peculiar in the injuries produced by railway accidents, whereas the most varied accidents may produce identical conditions. The author divides injuries of the back into three groups—viz., mere bruises, fractures, and marked nervous symptoms without any visible signs of injury. Many writers, however, restrict the term "railway spine" to the last class of injuries. Without external evidence there may be injury to the spinal contents, such as concussion, hæmorrhage into the spinal marrow or its coverings, later inflammatory changes, and still later degeneration. But nervous symptoms may exist when such results do not happen. The author observes: "For the benefit of our legal friends, let me say that by concussion of the brain or cord, we mean that the part suffers a loss or perversion of function which is of greater or lesser duration. This is sometimes accompanied by slight structural change. Very often, however, no visible change can be detected, and the alteration, whatever it may be, is molecular, and has been compared to the loss of its properties by a magnet when it is struck a blow."

Now so-called railway spine has been the subject of much difference of opinion and considerable controversy; doctors generally having little doubt on the matter, lawyers and railway directors generally professing a different opinion. Among eminent surgeons who have recognised spinal railway concussion is Mr. Erichson. That organic changes of the cord result from blows and shocks to the back is also maintained by Gowers, Edes, and Spitzka. There can be no manner of doubt that the severe shakings, even without a blow, consequent on railway accidents often result in a peculiar condition of the spinal cord. The rapidity of the movement causing the injury, the momentum of the person injured, the suddenness of its arrest, the helplessness of the sufferer, and the fear occasioned are all circumstances in railway accidents greatly increasing the severity of the injury to the nervous system. A person may be unaware that anything serious has happened, feeling, perhaps, only violently jolted and a little giddy and confused. After a while, however, he becomes excited and cannot sleep, and probably a day or two after he feels bruised all over, as if he had been through some violent labour. After a little time he finds himself unequal to exertion, or to attend to usiness. The thoughts become confused, the temper irritable, and the sleep disturbed. Such symptoms may pass off under complete rest for both mind and body, or other more pronounced signs may occur, as loss of freedom of movement and "straddling" gait. It may, however, be questioned if some cases characterised as above should not be regarded quite as much as railway brain cases, as railway spine cases. There is no reason, indeed rather the reverse, why concussion of both organs should not take place from the same violent shake. It is true that the spine is perhaps better protected from concussion than the brain, but there is every reason to believe that peculiar shakes may involve the latter organ as well as the former. In our opinion a better name for a certain number of these cases would be "shock to the nervous system," for they appear to be the result, not only of the mechanical shock, but also of the excessive mental and emotional excitement previously referred to. This class of cases includes some of the most difficult with which a medical man may have to deal. For he is called upon to distinguish between the malingerer and the honest sufferer. And even honest sufferers of a nervous, emotional, or hysterical temperament may unwittingly exaggerate the symptoms. Dr. Dercum says that as regards malingerers the expert must largely rely on his mother wit for the detection of the fraud. Distinction between actual and simulated organic disease can, as a rule, be readily made, though between simulated disease and hysteria the line is sometimes hard to draw. But the hysterical patient is generally emotional in the extreme, while the malingerer is a complacent, often argumentative liar, and occasionally displays a superficial familiarity with technical terms as though he had studied his part. Not unfrequently he contradicts himself, and can by leading questions be trapped into the grossest absurdities. But the investigation must be detailed and exhaustive, although after all it will be the "common-sense and native acumen which will guide him (the medical man)" in his conclusions. Stories have been told of persons being unable to attend to anything, and full of complaints, until they got a verdict, when they became suddenly rejuvenated. On the whole, however, we are inclined to think that such cases are not very common. As Spitzka observes, railway spine is undoubtedly the mask of a disseminated inflammatory trouble in a number of cases. When these patients survive recovery is slow, but ultimately may be perfect. Then the sufferer may be accused very unjustly of having malingered.

